# The Potential Use of Metabolic Cofactors in Treatment of NAFLD

**DOI:** 10.3390/nu11071578

**Published:** 2019-07-12

**Authors:** Adil Mardinoglu, Dilek Ural, Mujdat Zeybel, Hatice Hilal Yuksel, Mathias Uhlén, Jan Borén

**Affiliations:** 1 Science for Life Laboratory, KTH-Royal Institute of Technology, SE-17121 Stockholm, Sweden; 2 Centre for Host–Microbiome Interactions, Faculty of Dentistry, Oral & Craniofacial Sciences, King’s College London, London SE1 9RT, UK; 3 School of Medicine, Koç University, Istanbul 34450, Turkey; 4 Department of Gastroenterology and Hepatology, School of Medicine, Koç University, Istanbul 34450, Turkey; 5 Department of Molecular and Clinical Medicine, University of Gothenburg and Sahlgrenska University Hospital Gothenburg, 41345 Gothenburg, Sweden

**Keywords:** NAFLD, metabolic cofactors, l-carnitine, nicotinamide riboside, l-serine, *N*-acetyl-l-cysteine

## Abstract

Non-alcoholic fatty liver disease (NAFLD) is caused by the imbalance between lipid deposition and lipid removal from the liver, and its global prevalence continues to increase dramatically. NAFLD encompasses a spectrum of pathological conditions including simple steatosis and non-alcoholic steatohepatitis (NASH), which can progress to cirrhosis and liver cancer. Even though there is a multi-disciplinary effort for development of a treatment strategy for NAFLD, there is not an approved effective medication available. Single or combined metabolic cofactors can be supplemented to boost the metabolic processes altered in NAFLD. Here, we review the dosage and usage of metabolic cofactors including l-carnitine, Nicotinamide riboside (NR), l-serine, and *N*-acetyl-l-cysteine (NAC) in human clinical studies to improve the altered biological functions associated with different human diseases. We also discuss the potential use of these substances in treatment of NAFLD and other metabolic diseases including neurodegenerative and cardiovascular diseases of which pathogenesis is linked to mitochondrial dysfunction.

## 1. Introduction

Non-alcoholic fatty liver disease (NAFLD) is a consequence of the imbalance between deposition and removal of lipids from the liver [1,2,3]. The global prevalence of NAFLD continues to increase dramatically and has reached 25% at the population level [4,5,6,7]. NAFLD includes a spectrum of pathological conditions, ranging from simple steatosis to hepatic inflammation referred as non-alcoholic steatohepatitis (NASH), which can progress to cirrhosis and hepatocellular carcinoma (HCC) [8,9,10]. Even though hepatic steatosis and fibrosis are reversible conditions, decompensated cirrhosis is frequently associated with irreversible hepatic damage, and transplantation is considered to be the only possible treatment option. 

Oxidative stress and inflammatory responses have major role in the pathogenesis of NAFLD and its progression to NASH. NAFLD is also closely linked to complex metabolic conditions including obesity, type 2 diabetes mellitus (T2DM) and cardiovascular diseases (CVD) [11]. Considering the complexity associated with the drug development that can be used in effective treatment of the patients, supplementation of natural substances that can activate the altered metabolic pathways in NAFLD can be used in treatment of the patients. Supplementation of such metabolic cofactors may also improve the metabolic parameters in NAFLD patients and stop progression of the disease to severe form of the diseases including NASH, cirrhosis and HCC. To date, a number of metabolic cofactors have been supplemented to patients with different metabolic disorders and its positive effect has been shown in placebo controlled human clinical studies (Table 1 and Table S1).

The pathogenesis of NASH is a rather complex process consisting of lipid accumulation, hepatocellular injury, recruitment and activation of inflammatory and hepatic stellate cells, and fibrogenesis [8,10]. At earlier stages, lipid accumulation is a critical phase arising from increased dietary fatty acids and de novo lipogenesis and insufficient capacity for lipid removal by fatty acid oxidation and lipoprotein secretion. Elucidation of pathophysiological responses and cellular dysfunction induced by lipid accumulation is essential to develop efficient treatment strategies for NAFLD and other complex metabolic diseases including T2DM and CVD [12]. Such diseases have been frequently studied based on a single gene or a specific pathway. With the “omics” revolution that enable the generation of enormous amounts of data (i.e., genomics, transcriptomics, proteomics, lipidomics, metabolomics, fluxomics, epigenetics, and metagenomics), identification of key genes and essential pathways driving steatosis, steatohepatitis and fibrosis in NAFLD is possible using a holistic systems biology approach [13,14]. However, analyzing multi-layer omics data to understand the pathophysiology is a challenging task. 

Systems biology enables the integration and analysis of multi-layer omics data to make predictions that can be experimentally tested [15,16,17,18,19,20]. This integrative approach has successfully been used in various contexts in medical research over the last decade. We have recently performed systems level analysis by combining clinical studies with stable isotopes, in-depth multi-omics profiling and personalized network analysis, and revealed the underlying mechanisms of NAFLD to developing novel strategies for its prevention and treatment [16,20,21]. Our integrated analysis indicated that there is an augmented requirement for Nicotinamide adenine dinucleotide (NAD)+ and reduced glutathione (GSH) in patients with NAFLD. This observation was further validated using liver transcriptomics and plasma metabolomics data. We also performed a mouse supplementation study with the precursors for NAD+ (Nicotinamide riboside [NR]) and GSH (l-serine [serine] and *N*-acetyl-l-cysteine [NAC]), and a proof of concept human supplementation study with serine to decrease the hepatic steatosis. 

**Figure 1:**
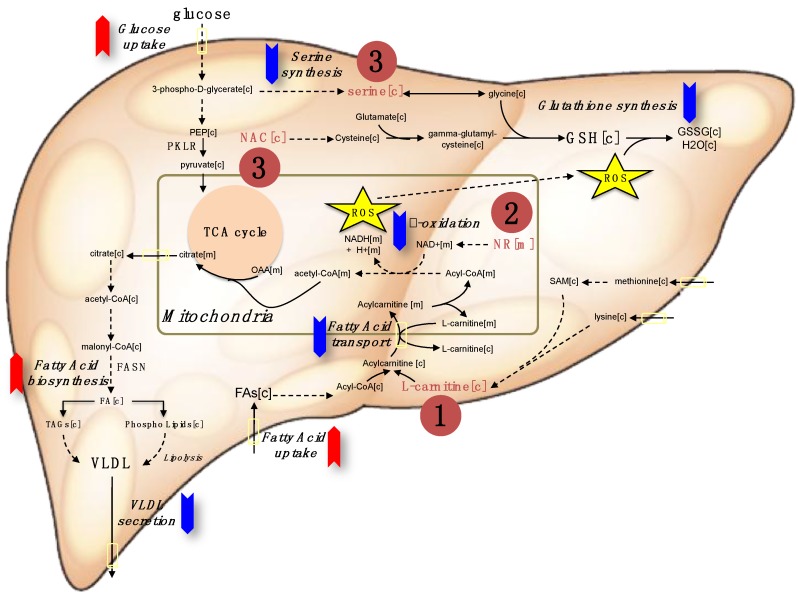
The red and blue arrows indicate the upregulation and down regulation of metabolic pathways in NAFLD, respectively. Metabolic cofactors can be supplemented to treat such metabolic abnormalities for effective treatment of the patients. The three-step strategy can be applied by supplementing (1) l-carnitine to enhance the transport of fatty acids across the mitochondrial membrane, (2) the NAD^+^ precursor nicotinamide riboside to enhance the β-oxidation of fatty acids in mitochondria, and the (3) glutathione (GSH) precursors including serine and *N*-acetyl-l-cysteine (NAC) to form GSH that is required to protect liver against free radical-mediated oxidative stress generated by the increased β-oxidation of fatty acids in the mitochondria. TCA: The citric acid cycle, VLDL: Very-low-density lipoprotein, ROS: Reactive oxygen species. PKLR: pyruvate kinase L/R, FASN: Fatty acid synthase.

Our analysis indicated that a three-step strategy including (i) increasing mitochondrial fatty acid uptake, (ii) increasing mitochondrial fatty acid oxidation, and (iii) increasing the availability of GSH can be applied to decrease the amount of hepatic steatosis in NAFLD patients. Single or combined metabolic cofactors can be supplemented to boost these metabolic processes altered in NAFLD. Supplementation of (i) l-carnitine (carnitine) may stimulate the transfer of fatty acids from cytosol to mitochondria, (ii) NR may provide required amount of NAD+ which is essential for mitochondrial fatty acid oxidation, and (iii) serine and NAC may increase the level of GSH in the cells (Figure 1). Here, we conducted a literature review about the use of individual metabolic cofactors including carnitine, NR, serine and NAC, in human clinical studies to treat different disorders. We also provided a guideline for use of these metabolic cofactors by reviewing different human clinical studies and observed that careful screening of the patients during human clinical studies may decrease the risk associated with the combination of metabolic cofactors.

## 2. Potential Risks and Benefits of Metabolic Co-Factors

Chronic metabolic disorders and aging are commonly associated with reduced plasma NAD^+^ and GSH levels [22,23,24]. Since hepatocellular synthesis of GSH by de novo or by the salvation pathways are required; intracellular GSH levels cannot be increased by simply providing GSH supplements itself. Based on our integrative analysis, we found that the level of GSH is not enough to maintain and regulate the thiol redox status of the liver in subjects with high hepatic steatosis at fasting stage due to depletion of glycine and serine [25]. It has been shown that serine synthesis is downregulated in NAFLD patients [25] and supplementation of serine improved liver tissue function and decreased the liver steatosis in a proof of concept human study [21]. 

We aimed to decrease the level of liver fat content in NAFLD patients by increasing the hepatic levels of pivotal metabolic cofactors via simultaneous dietary supplementation of carnitine, NR, serine, and NAC. The study is based on a three-step strategy to increase the amount of fat oxidized in their liver (Figure 1). First, we included carnitine, which is transported by carnitine palmitoyltransferase (CPT) I and II and generates a long chain acetyl carnitine ester to facilitate the transport of fatty acids across the mitochondrial membrane. In addition to that, carnitine is important for exchanging acetyl groups and stabilization of acetyl-CoA and coenzyme A. Second, we included NR, a precursor of NAD^+^, to boost the level of hepatic mitochondrial β-oxidation of fatty acids and activate mitochondria. Decreased electron transport chain function combined with impaired rates of fatty acid β-oxidation leads to the accumulation of incomplete products of β-oxidation, which combined with increased levels of reactive oxygen species (ROS) contribute to insulin resistance. Finally, we included the two GSH precursors serine and NAC to increase GSH levels in the hepatocytes. Increased GSH levels may protect against free radical-mediated oxidative stress generated by the increased β-oxidation of fatty acids in mitochondria. Combined metabolic cofactors may concomitantly stimulate these three different metabolic pathways and activate the liver tissue metabolism in NAFLD patients.

Metabolic cofactors including carnitine, NR, serine, or NAC have been supplemented individually for treatment of different disorders (Table 1 and Table S1). Neither of them is known to have significant side effects or toxicity. Human clinical studies related to these metabolic cofactors as well as pharmacological data on each metabolic-cofactor is summarized below.

### 2.1. l-Carnitine 

Carnitine is a white crystalline, hygroscopic powder and readily soluble in water and hot alcohol, and is insoluble in acetone. The absolute bioavailability of carnitine is ~15–16%. The mean distribution half-life is ~0.6 h and the mean apparent terminal elimination half-life is 17.4 h. Total body clearance of carnitine (Dose/AUC including endogenous baseline concentrations) is a mean of 4.00 L/h. Carnitine is not bound to plasma protein or albumin when tested at any concentration [26]. 

The major metabolites are trimethylamine *N*-oxide, excreted primarily in urine (8% to 49% of the administered dose) and [3H]-γ-butyrobetaine, excreted primarily in feces (0.44% to 45% of the administered dose). Urinary excretion of carnitine is about 4 to 8% of the dose. Fecal excretion of total carnitine is less than 1% of the administered dose [27].

Carnitine is a naturally occurring substance required in mammalian energy metabolism. It is a carrier molecule that facilitates the transport of long-chain fatty acids across the inner mitochondrial membrane and delivers substrate for oxidation of fatty acids and subsequent energy production. Carnitine supplementation is recommended to the patients with inborn errors of metabolism and patients undergoing hemodialysis for kidney disease.

Carnitine deficiency is characterized with very low carnitine levels in plasma and tissues and may be either primary or secondary. Primary carnitine deficiency is an autosomal recessive disorder caused by a deficiency in the plasma membrane carnitine transporter and leads to urinary carnitine wasting [28]. SLC22A5 mutations can also affect carnitine transport and decrease plasma carnitine levels. In primary systemic deficiency, the clinical complications are associated with the recurrent episodes of Reye-like encephalopathy, hypoketotic hypoglycemia, and/or cardiomyopathy. Associated symptoms also include hypotonia, muscle weakness, and failure to thrive. In some patients, particularly those presenting with cardiomyopathy, carnitine supplementation may rapidly alleviate signs and symptoms. Secondary carnitine deficiency is associated with inadequate carnitine intake, decreased carnitine synthesis due to liver disorders, loss of carnitine during diarrhea, diuresis, or hemodialysis [29].

Carnitine is used in the treatment of primary and secondary carnitine deficiency [30]. It may also be recommended to the patients taking certain drugs (such as valproic acid for seizures or antibiotics for tuberculosis), or during medical procedures (hemodialysis for kidney disease) that deplete the body’s carnitine deficiency [30].

Carnitine has also been supplemented to patients with heart failure, ischemic heart diseases, peripheral arterial diseases, HIV, male infertility, anorexia, chronic fatigue syndrome, and fatigue associated with chronic diseases and chronic obstructive pulmonary disease (Table 1). It is also used as a replacement supplement in strict vegetarians, dieters, and low-weight or premature infants. In athletes, carnitine has been used to improve performance, although the beneficial effects in athletes are not consisted in all studies. 

#### 2.1.1. Dosage

The recommended daily dosage of carnitine is 1 to 3 g, but higher doses have been used in clinical studies (see Table 1). Numerous studies have been performed:

⮚ In patients with carnitine depletion in peripheral blood mononuclear cells, carnitine has been supplemented at a dose of 6 g/day for 2 weeks [31].
⮚ Several studies have tested if carnitine supplementation promotes weight loss in obese subjects (4 g/L, 8 weeks) [32].
⮚ Efficacy and effectiveness of carnitine supplementation for cancer-related fatigue has been analyzed in a systematic literature review and meta-analysis. Nine studies used a dose between 2 to 6 g per day [33].
⮚ Impact of carnitine supplementation on plasma lipoprotein(a) concentrations have been analyzed in a recent systematic review and meta-analysis of human clinical trials. Studies used 2–4 g/day [34].
⮚ A systematic review was conducted to determine the effects of carnitine on all-cause mortality and cardiovascular morbidities in the setting of acute myocardial infarction (meta-analysis of five controlled trials, *n* = 3108). There were no significant differences between the effects of daily carnitine supplementation of 2 g and 6 g on heart failure, unstable angina, or myocardial reinfarction [35].
⮚ The effect of carnitine supplementation on the regression of NASH was evaluated in 74 patients with a clinical and pathologic diagnosis of NASH [36]. The study subjects were randomly allocated to the placebo or to the carnitine (2 g per day divided into two equal doses for 24 weeks) groups. At the end of the study, carnitine-treated patients showed significant improvements in AST, ALT, gamma-GT, total cholesterol, LDL-cholesterol, HDL-cholesterols, triglycerides, glucose, HOMA-IR, C-reactive protein, TNF-alpha, and histological scores. Thus, carnitine supplementation reduced inflammation, and improved liver function, glucose plasma level, lipid profile, HOMA-IR, and histological manifestations of NASH.

#### 2.1.2. Safety Aspects

Carnitine is usually well-tolerated and the FDA granted carnitine with Generally Recognized as Safe (GRAS) status. Various mild gastrointestinal complaints have been reported during the long-term administration of oral l- or d, carnitine; including transient nausea and vomiting, abdominal cramps, and diarrhea. At doses of approximately 3 g/day, carnitine supplements can cause nausea, vomiting, abdominal cramps, diarrhea, and a “fishy” body odor. Rare side effects include muscle weakness/mild myasthenia in uremic patients and seizures in those with seizure disorders.

Gastrointestinal adverse reactions with carnitine may be avoided by a slow consumption of the solution or by a greater dilution. Decreasing the dosage often diminishes or eliminates drug-related patient body odor or gastrointestinal symptoms when present. Tolerance should be monitored very closely during the first week of administration, and after any dosage increases. 

It has been reported that the intestinal bacteria may metabolize carnitine to form a substance called Trimethylamine-*N*-oxide (TMAO) that might increase the risk of cardiovascular disease [37]. This effect appears to be more pronounced in people who consume meat than in vegans or vegetarians. The implications of these findings are not well understood and require more research.

There have been no reports of toxicity from carnitine over dosage. Carnitine is easily removed from plasma by dialysis. Large doses of carnitine may cause diarrhea. Pregnancy risk category of carnitine is B and it should be used during pregnancy only if it is clearly needed. Drug interaction with carnitine is very rare but it may decrease the effectiveness of the thyroid hormone and increase the effects of warfarin. 

### 2.2. Nicotinamide Riboside

NAD is an endogenous substance that is involved in several important cell functions such as signal transduction, DNA repair, and post-translational protein modifications. NAD-consuming activities and cell division necessitate ongoing NAD synthesis, either through a de novo pathway that originates with tryptophan or via salvage pathways from three NAD^+^ precursor vitamins, NR, nicotinamide, and nicotinic acid. In animals, NAD generation is vital, since it is linked to several redox reactions in the body. NAD plays a central role in fatty acid metabolism, energy metabolism, and oxidative phosphorylation and is a key component of many metabolic pathways for carbohydrates, lipids, and amino acids.

NR is in wide use as an NAD+ precursor vitamin. It is available as an over-the-counter dietary supplement. The therapeutic potential of NAD has been investigated in several clinical conditions (e.g., aging), improving exercise performance, assisting weight loss and treating chronic fatigue syndrome [38]. Its effect has also been tested in improving depression, cognitive function, as well as treating patients with dyslipidemia, diabetes, Parkinson’s disease, Alzheimer’s disease, and dementia (Table 1). 

#### 2.2.1. Dosage

The daily dose of NR is 500–2000 mg in previously performed human clinical studies (Table 1). Numerous studies have been performed:

⮚ Trammell et al. determined the time and dose-dependent effects of NR on blood NAD^+^ level in humans [39]. They reported that human blood level of NAD^+^ can rise as much as 2.7-fold with a single oral dose of NR in a pilot study. They also demonstrated that single doses of 100, 300, and 1000 mg of NR produce dose-dependent increases in the blood NAD^+^ metabolome in the first clinical trial of NR pharmacokinetics in humans.
⮚ Airhart et al. recently reported an open-label, non-randomized study of the pharmacokinetics of NR and its effects on blood NAD^+^ levels [40]. In eight healthy volunteers, 250 mg NR was orally administered on days 1 and 2, then uptitrated to peak dose of 1000 mg twice daily on days 7 and 8. On the morning of day 9, subjects completed a 24-hour pharmacokinetic study after receiving 1000 mg NR at *t* = 0. They analyzed whole-blood levels of NR, clinical blood chemistry, and NAD^+^ levels and reported that oral NR was well tolerated with no adverse events. Significant increases comparing baseline to mean concentrations at steady state were observed for both NR (*p* = 0.03) and NAD^+^ (*p* = 0.001); the latter increased by 100%. Absolute changes from baseline to day 9 in NR and NAD^+^ levels correlated highly (*R*^2^ = 0.72, *p* = 0.008). The authors concluded that NR increases circulating NAD^+^ in humans and it may be used in treatment of patients with diseases associated to mitochondrial dysfunction [40].

#### 2.2.2. Safety Aspects

In 2016, the FDA granted NR with GRAS status on the basis of existing clinical study, which showed that the “no observed adverse effect level (NOAEL) was 300 mg/kg/day.” NR seems safe for most people when used appropriately and short-term, up to 12 weeks [41]. It is also approved as a food ingredient in enhanced water products, protein shakes, nutrition bars, and chewing gum at no more than 0.027% by weight.

There was no mortality at an oral dose of 5000 mg/kg. Based on the results of a 14-day study, a 90-day study was performed comparing NR at 300, 1000, and 3000 mg/kg/day to an equimolar dose of nicotinamide at 1260 mg/kg/day as a positive control. Results from the study show that NR had a similar toxicity profile to nicotinamide at the highest dose tested. The lowest observed adverse effect level for NR was 1000 mg/kg/day, and the no observed adverse effect level was 300 mg/kg/day.

Flushing is the most sensitive side effect of nicotinic acid supplementation. After ingestion of supplemental nicotinamide, no cases of flushing or glucose intolerance have been reported and only one case of hepatitis was reported following the ingestion of greater than 3 g/day for several days. Such side-effects have not been reported for supplementation of NR. NR was not genotoxic but data on its use during pregnancy and breast-feeding is inadequate. There is no known drug interaction with the supplementation of NR.

### 2.3. l-Serine 

Serine is a non-essential amino acid; however, under certain circumstances, vertebrates cannot synthesize it in sufficient quantities to meet necessary cellular demands. Serine is biosynthesized in the mammalian system from 3-phosphoglycerate and serves as a precursor for the synthesis of the amino acids including glycine and cysteine. Physiologically, it has a variety of roles, perhaps most importantly as a phosphorylation site in proteins. Mutations in the metabolic enzymes that synthesize serine have been implicated in various human diseases. 

#### Dosage

The FDA determined serine with GRAS status and it appears to be neuroprotective. The daily dose of serine is 1–30 g in previous human clinical studies (Table 1), but higher doses have been used in some clinical studies (Table 1). Numerous studies have been performed including:

⮚ Serine supplementation (three daily doses of 5 g of serine [i.e., 190 mg/kg]) has also been shown to be safe even in pregnancy, as shown by in pre- and postnatal treatment of 3-phosphoglycerate-dehydrogenase deficiency [42,43].
⮚ Hereditary sensory and autonomic neuropathy type 1 (HSAN1) is a disorder caused by missense mutations in the enzyme serine palmitoyltransferase (SPT) [44]. Subjects received daily supplements of powdered serine (mixed in water) on a low- or high-dose schedule (200 or 400 mg/kg body weight, respectively; *n* = 7 per group). Results showed that an altered substrate selectivity of the mutant SPT is key to the pathophysiology of HSAN1 and raise the prospect of serine supplementation as a first treatment option for this disorder [44].
⮚ In our previous study, we assessed the effect of dietary supplementation with serine (200 mg/kg per day) for 2 weeks on fatty liver and fasting levels of plasma markers of liver functions in six obese subjects with NAFLD. Our analysis showed that supplementation of serine improved markers of liver tissue function and significantly decreased liver fat [21].
⮚ Fridman et al. performed a randomized, double-blind, placebo-controlled trial (*n* = 18) to evaluate the efficacy and safety of serine treatment for adults with hereditary sensory and autonomic neuropathy type 1 (HSAN1). The study subjects were randomized to serine (400 mg/kg/day) or placebo for one year. All participants received serine during the second year. Analysis of vital signs, physical examination findings, and clinical laboratory examinations did not reveal adverse effects of serine. Thus, long-term serine supplementation did not reveal adverse effects of serine [45].

### 2.4. N-Acetyl-L-Cysteine

NAC is a white to white with light yellow cast powder, and has a pKa of 9.5 at 30 °C. NAC is stable in gastric and intestinal fluids and rapidly absorbed after oral administration. It is not affected by food intake. It reaches peak plasma concentration in 30–60 min after application. The distribution volume (Vd) is between 0.33 and 0.47 L/kg, which is evident in extracellular fluids and passes primarily to the lung, kidney and liver. After oral administration, 48% of the amount passed to the blood is determined in the lungs. The rate of binding to plasma proteins is about 50%. NAC is extensively metabolized in liver, and 22–30% is excreted in urine in the form of sulfate and taurine. NAC has a half-life of 5.6–6 h in adults.

NAC is the *N*-acetyl derivative of the amino acid l-cysteine and it is an essential precursor in the formation of the antioxidant glutathione within the human body. Hence, administration of NAC replenishes glutathione stores.

Glutathione may act as an endogenous neuromodulator, modulate the redox state of the *N*-methyl-d-aspartate receptor complex, and activate ionotropic receptors that are different from any other excitatory amino acid receptor, which may constitute glutathione receptors, potentially making it a neurotransmitter [46]. Since NAC is a prodrug of glutathione, it may modulate some/all of the glutathione receptors. It has potential to confer antioxidant effects and may reduce free radicals. NAC also possesses some anti-inflammatory effects possibly via inhibiting NF-κB and modulating cytokine synthesis [47]. 

NAC has two approved indications: (i) treatment of paracetamol (acetaminophen) overdose associated with liver damage and (ii) removal of thick mucus in individuals with cystic fibrosis or chronic obstructive pulmonary disease. NAC has also been tested for contrast induced nephropathy, infertility, cystic fibrosis, ischemic heart diseases, HIV, hypercholesterolemia, and schizophrenia (Table S1). It is beneficial effect has been reported in any kind of acute hepatic failure and treatment of liver diseases as discussed below.

#### 2.4.1. Dosage

The daily dose of NAC is 1–5 g in previous human clinical studies (Table S1), but higher doses have been used in different clinical studies (Table S1).

NAC is available as intravenous (IV) and oral formulations. The IV injection and inhalation preparations are, in general, prescription only, whereas the oral solution and the effervescent tablets are available over the counter in many countries including the United States. Daily dose of NAC is 200 mg capsules taken 3 times a day (morning, lunch, evening) or a single dose of 600 mg (3 capsules) in the evening. In paracetamol poisoning, the loading dose is ~140 mg/kg, and the maintenance dose is 70 mg/kg every 4 h (17 doses). Numerous studies have been performed:

⮚ In early psychosis, NAC was administered at a dose of 2700 mg/day for 6 months in a double-blind placebo-controlled trial [48].
⮚ NAC was administered to enhance performance of elite sport. A recent systematic review of the literature evaluated the effect of NAC supplementation. The typical daily dose of NAC reported was 5.8 g/day; with a range between 1.2 and 20.0 g/day [49].
⮚ The effect of NAC supplementation on oxidative stress status and alveolar inflammation was analyzed in a double-blind, randomized clinical trial using a dose of 1800 mg/day for 4 months in people exposed to asbestos [50].
⮚ NAC was administered in oral doses of 6000–8000 mg daily for several months in HIV-infected patients. It had a good safety profile and minimal adverse effects [51].
⮚ NAFLD patients (*n* = 30) were randomly selected to receive either NAC (600 mg per 12 h) or vitamin C (1000 mg per 12 h) [52]. Liver function tests (ALT, AST and ALP) were measured as well as the grade of steatosis, the pattern of its echogenicity, the span of the liver and the spleen, and the portal vein diameter before the intervention. Patients were followed up using the same method of evaluation repeated in the first, second, and third months. NAC resulted in a significant decrease of serum ALT after three months, compared to vitamin C. This effect was independent of the grade of steatosis in the initial diagnosis. It has been reported that NAC significantly decrease the span of the spleen and it can be used to improve liver function in patients with NAFLD [52].
⮚ The therapeutic effect of NAC in the treatment of NASH was investigated in 35 patients diagnosed with NASH based on liver biopsy. Patients were divided into two groups: the first (18 patients) was administered NAC 600 mg/day orally for 4 weeks, while the control group (17 patients) was followed up without therapy. Results did not show improved liver function in this study. It has been reported that the daily amount of glutathione synthesis in humans is 10–15 g and most of the sources of this are provided from the natural sources of the organism. Therefore, the authors hypothesized that the lower dosage of NAC (600 mg/day) might not affect glutathione synthesis to a great extent [53].
⮚ To test whether glutathione deficiency occurs due to the diminishd synthesis and contributes to oxidative stress, eight elderly (60–75 years) and eight younger (30–40 years) subjects received stable-isotope infusions of [^2^H(_2_)]glycine, after which red blood cell (RBC) glutathione synthesis and concentrations, plasma oxidative stress, and markers of oxidant damage were measured. Results showed that glutathione deficiency in elderly humans occurs because of a marked reduction in synthesis, and that dietary supplementation with the two glutathione precursors cysteine (as *NAC*) and glycine fully restores glutathione synthesis and concentrations and lowers levels of oxidative stress and oxidant damages [54].

#### 2.4.2. Safety Aspects

Side effects occur rarely with NAC usage. Even at very high doses, serious adverse events or signs of intoxication have not been observed. Most common adverse reactions (incidence greater than 2%) are rash, urticaria/facial flushing, pruritus, nausea, and vomiting. Adverse effects for oral formulations of NAC have been reported to include nausea, vomiting, rash, and fever.

Although unclear, there was a trend of increasing side effects with increasing doses and IV usage of NAC compared with placebo. Anaphylactic reactions, i.e., pruritus, rash, angioedema, bronchospasm, tachycardia, and hypotension have been previously reported to occur within 30 min after IV loading dose of NAC in ~3–6% of people. Hypersensitivity reactions, including generalized urticaria, have been observed in patients receiving oral NAC for acetaminophen overdose, but anaphylaxis has rarely been reported with oral administration.

Occasionally, severe and persistent vomiting occurs as a symptom of acute acetaminophen overdose. Treatment with NAC may aggravate the vomiting and increase the risk of upper gastrointestinal hemorrhage in at risk patients (e.g., those with esophageal varices, peptic ulcers, etc.).

Large doses in a mouse model showed that NAC could potentially cause damage to the heart and lungs. It has been shown that NAC was metabolized to *S*-nitroso-*N*-acetylcysteine, which increased blood pressure in the lungs and right ventricle of the heart (pulmonary artery hypertension) in mice treated with NAC. The effect was similar to that observed following a three-week exposure to an oxygen-deprived environment (chronic hypoxia). The authors also found that *S*-nitroso-*N*-acetylcysteine induced a hypoxia-like response in the expression of several important genes both in vitro and in vivo.

No specific antidote is currently available; supportive and symptomatic treatments are performed. NAC is contraindicated in patients with previous allergic/anaphylactoid reaction to NAC. Patients with acute asthma attacks can also not use NAC. Pregnancy risk category of NAC is B. There is insufficient data on the usage in pregnancy and lactation and it should only be used if it is necessary. Drug interaction with NAC is very rare. Minor interactions have been reported with parenteral nitroglycerine and oral forms of nitrates. NAC has no effect on the use of vehicles and machinery. 

## 3. Conclusions

Here, we reviewed the dosage and known safety of carnitine, NR, serine, and NAC in different human clinical trials with a special emphasis on human safety. Three of the compounds are found naturally as constituents in food, except for NAC, which is a drug, despite its primary metabolite being cysteine. These metabolic cofactors can be tested in future and clinical implications in single or combined form for treatment of different diseases.

The concept that substrate deficiencies for metabolic enzymes underlie the progression of and may even be the causes of human diseases, and it has rarely been mechanistically explored for different diseases. Based on our integrative systems level analysis, we suggested the use of carnitine, NR, serine, and NAC for effective treatment of NAFLD. Of note, following the failure of recent clinical trials targeting single molecule or pathway; current NASH studies show that the trend towards targeting multiple pathways employing combination therapeutics. This is in line with changing our understanding of NASH pathogenesis from double-hit to multiple-hit hypothesis [55]. Therefore, a combination of supplements targeting several NASH pathways would probably be more appropriate. 

From a toxicological point of view, using nutritional substances rarely causes safety issues in humans. The “safe doses” for most nutritional/food substances are already known in humans and, unlike most pharmaceuticals, there is often a very large margin to toxicity using nutritional compounds. As an example, the dose at which vitamin B_12_ becomes toxic for humans is over 100-fold higher than the recommended daily intake. The reason for this low toxicity of nutritional substances is because they are generally directly or indirectly metabolized via cellular metabolic pathways. The products are easily dealt with either by shuffling substrate-overload to other metabolic pathways, or used as building blocks of other compounds (such as glycogen, fat, proteins), or excreted from the cell or body. 

Despite this, many calls to national poison centers occur every year due to ingestion of high doses of vitamins and minerals, especially for the fat-soluble vitamins A, D, and iron. Regarding pharmaceuticals, with the exception of some antibiotics, very few drugs are given in gram (g) doses. Most drugs are metabolized in the liver in order to aid excretion from the body. Almost all medical drugs are foreign to the body, and their individual structures and use are largely directed to alteration of an enzyme or receptor activity, before being excreted from the body. Hence, during the supplementation of these metabolic cofactors in human clinical trials, patients should be screened carefully to avoid potential side effects on liver, heart and kidney functions.

We demonstrated that simultaneous supplementation of combined metabolic cofactors may improve the efficacy of the intervention in patients with NAFLD and decreases liver fat [21]. Considering that NAFLD, obesity, type 2 diabetes, neurodegenerative diseases, and cardiovascular diseases are common conditions that regularly co-exist and act synergistically [56]. Furthermore, it has a similar pathogenesis with alcoholic fatty liver disease [57]; such metabolic cofactors can also be used in the treatment of the subjects with such disorders after testing its effect in placebo-controlled human clinical studies.

## Figures and Tables

**Table 1:** The dosages of the Nicotinamide Riboside, l-serine, and l-carnitine used in previous human trials. The data retrieved from https://clinicaltrials.gov. The dosage of the *N*-acetyl-l-cysteine is provided in Table S1.

NCT Number	Title	Dosage	Conditions	Phases
	**Nicotinamide Riboside (All Studies)**			
NCT03423342	Nicotinamide Riboside in Systolic Heart Failure	500–2000 mg/day	Heart Failure, Systolic	Phase 1|Phase 2
NCT02689882	Pharmacokinetic Study of Nicotinamide Riboside	500–2000 mg/day	Metabolic Disturbance	Phase 1
NCT03432871	Nicotinamide Riboside and Mitochondrial Biogenesis	10 mg/kg/day	Mitochondrial Diseases	Not Applicable
NCT02835664	Nicotinamide Riboside and Metabolic Health	1000 mg/day	Obesity|Insulin Resistance	Not Applicable
NCT02812238	Study to Evaluate the Effect of Nicotinamide Riboside on Immunity	1000 mg/day	Atherosclerosis|Diabetes|Coronary Artery Disease	Phase 2
NCT03685253	Nicotinamide Riboside for Diabetic Neuropathy	1000 mg/day	Diabetic Neuropathy Peripheral	Phase 1|Phase 2
NCT02921659	Safety & Efficacy of Nicotinamide Riboside Supplementation for Improving Physiological Function in Middle-Aged and Older Adults	1000 mg/day	Aging	Phase 1|Phase 2
NCT03743636	Nicotinamide Riboside With and Without Resveratrol to Improve Functioning in Peripheral Artery Disease	1000 mg/day	Peripheral Artery Disease	Phase 3
NCT03818802	Effects of Vitamin B3 Derivative Nicotinamide Riboside (NR) in Bone, Skeletal Muscle and Metabolic Functions in Aging	1000 mg/day	Healthy Elderly Volunteers	Not Applicable
NCT03579693	Trial of Nicotinamide Riboside and Co-enzyme Q10 in Chronic Kidney Disease	1200 mg/day	Chronic Kidney Disease|Sarcopenia|Frailty	Phase 2
NCT03727646	Nicotinamide Riboside in LVAD Recipients	2000 mg/day	Heart Failure	Early Phase 1
NCT03789175	Nicotinamide Riboside on Mitochondrial Function in Li-Fraumeni Syndrome	500–2000 mg/day	Cancer|Skin Fibroblasts|Muscle Weakness	Phase 1|Phase 2
NCT03501433	Effects of Nicotinamide Riboside on Metabolism and Vascular Function	500 mg/day	Aging|Lipemia	Not Applicable
NCT03821623	Nicotinamide Riboside for Treating Elevated Systolic Blood Pressure and Arterial Stiffness in Middle-aged and Older Adults	1000 mg/day	Hypertension|Aging	Phase 2
NCT03912220	Evaluation of Nicotinamide Riboside in Prevention of Small Fiber Axon Degeneration and Promotion of Nerve Regeneration	1800 mg/day	Small Fiber Neuropathy	Phase 2
NCT03754842	Effect of Nicotinamide Riboside and Pterostilbene Supplementation on Muscle Regeneration in Elderly Humans	1000 mg/day	Muscle Injury	Not Applicable
NCT03151707	The Effects of Nicotinamide Riboside Supplementation on NAD+/NADH Ratio and Bioenergetics	1000 mg/day	Healthy	Phase 4
NCT03565328	The Effect of Nicotinamide Riboside on Skeletal Muscle Function in Heart Failure Subjects	500–2000 mg/day	Heart Failure	Phase 2
NCT03642990	NR in Chemo-induced Peripheral Neuropathy	300–1000 mg/day	Chemotherapy-induced Peripheral Neuropathy	Phase 2
NCT03951285	Nicotinamide Riboside and Mitochondrial Metabolism	250–1000 mg/day	Obesity	Not Applicable
NCT03562468	A Study by ChromaDex to Assess the Effects of TRU NIAGEN on Cognitive Function, Mood and Sleep in Older Adults	300–1000 mg/day	Cognitive Function|Mood|Sleep	Not Applicable
NCT03962114	Effects of Vitamin B3 in Patients With Ataxia Telangiectasia	25 mg/kg/day	Ataxia Telangiectasia|ATM Gene Mutation	Phase 2
NCT02300740	Pharmacokinetic Analysis of Nicotinamide Riboside	500–1000 mg/day	Healthy Participants	Early Phase 1
NCT02712593	A Study Investigating the Effects of Niagen in Healthy Adults.	100–1000 mg/day	Bioavailability	Phase 2
NCT02721537	Use of 31P MRS to Assess Brain NAD+ in Healthy Current and Former Collegiate Athletes	750 mg/day	Concussion, Mild	Not Applicable
NCT03176628	Pharmacokinetics, Pharmacodynamics and Safety of Basis in Acute Kidney Injury Study	500–2000 mg/day	Acute Kidney Injury	Not Applicable
NCT02303483	The Effect of Vitamin B3 on Substrate Metabolism, Insulin Sensitivity, and Body Composition in Obese Men	2000 mg/day	Obese	Not Applicable
NCT02191462	A Study of the Pharmacokinetics of Three Dosages of Niagen in Healthy Subjects	100–1000 mg/day	Pharmacokinetics	Phase 1
NCT02950441	Nicotinamide Adenine Dinucleotide and Skeletal Muscle Metabolic Phenotype	1000 mg/day	Aging	Phase 2
NCT03568968	A Randomized Controlled Trial of Nicotinamide Supplementation in Early Parkinson’s Disease	1000 mg/day	Parkinson Disease	Not Applicable
NCT02942888	The Effects of Nicotinamide Adenine Dinucleotide (NAD) on Brain Function and Cognition	250–1000 mg/day	Mild Cognitive Impairment|NAD	Not Applicable
NCT02678611	A Study to Evaluate Safety and Health Benefits of Basis Among Elderly Subjects.	250–500 mg/day	Safety: Healthy Subjects	Phase 1
	**l-serine (All Studies)**			
NCT02528994	Short Term Dietary Serine Supplementation and Circulating Serine Levels	6–48 g/day	Bioavailability	Not Applicable
NCT03062449	Phase IIa l-serine Trial for eAD	30 g/day	Alzheimer Disease	Phase 2
NCT01835782	Determining the Safety of l-serine in ALS	1–30 g/day	Amyotrophic Lateral Sclerosis (ALS)	Phase 1|Phase 2
NCT03580616	Tolerability and Efficacy of l-Serine in Patients With Amyotrophic Lateral Sclerosis (ALS)	30 g/day	Amyotrophic Lateral Sclerosis	Phase 2
NCT01733407	l-Serine Supplementation in Hereditary Sensory Neuropathy Type 1	0.4 g/kg/day	Hereditary Sensory and Autonomic Neuropathy Type I	Phase 1|Phase 2
**NCT02599038**	Serine Supplementation for Obese Subjects With Fatty Liver Disease	0.2 g/kg/day	Non-alcoholic Fatty Liver Disease	Phase 1|Phase 2
	**l-carnitine (Completed Studies)**			
NCT03953248	l-Carnitine as an Adjuvant Treatment in Acute Phosphide Poisoning (LC)	1000 mg/8 h	Toxicity	Early Phase 1
NCT02281253	Effects of a Bakery Product Enriched With Fibre and l-carnitine on Insulin Resistance in Patients With Metabolic Syndrome	2325 mg/day	Metabolic X Syndrome|Overweight|Dyslipidemias	Not Applicable
NCT03008356	l-carnitine for Fatigue in COPD	2000 mg/day	Copd|Fatigue	Phase 2|Phase 3
NCT00809042	Combination Therapy of Hydroxyurea With l-Carnitine and Magnesium Chloride in Thalassemia Intermedia	250 mg/day	Œ≤-Thalassemia Intermedia	Phase 2
NCT00386971	Effects of l-Carnitine on Postprandial Clearance of Triglyceride-rich Lipoproteins in HIV Patients on HAART	3000 mg/day	Hyperlipidemia|HIV Infections	Not Applicable
NCT03476356	l-Carnitine and Clomiphene Citrate for Induction of Ovulation in Women With Polycystic Ovary Syndrome	3000 mg/day	Polycystic Ovary Syndrome	Not Applicable
NCT01580553	The Clinical Study of the Efficacy and Safety of l-Carnitine Injection in Treatment of Heart Failure	1000 mg/day	Heart Failure,	Phase 2|Phase 3
NCT03630341	Adding l-Carnitine to Clomiphene Citrate for Induction of Ovulation in Women With Polycystic Ovary Syndrome	1000 mg/day	Polycystic Ovary Syndrome	Phase 4
NCT00247975	Ability of l-carnitine to Prevent Heart Damage in Breast Cancer Patients Receiving Anthracycline Chemotherapy	3000 mg/day	Heart Failure	Phase 2|Phase 3
NCT00822172	Evaluation of Cilostazol in Combination With l-Carnitine	2000 mg/day	Peripheral Vascular Disease	Phase 4
NCT01769157	Effects of l-carnitine on Hypothyroidism	1980 mg/day	Hypothyroidism	Phase 4
NCT01232907	The Effects of l-carnitine on Fatigue in Spinal Cord Injury	1980 mg/day	Spinal Cord Injury (SCI)	Phase 2
NCT00841295	Effects of Parenteral l-carnitine Supplementation in Premature Neonates	10 mg/kg/day	Complication of Prematurity	Not Applicable
NCT02692235	Carnitine Supplementation and Skeletal Muscle Function	1500 mg/day	Sarcopenia	Phase 3
NCT01149525	Efficacy of l-carnitine Versus Placebo in the Treatment of Fatigue in Multiple Sclerosis	4000 mg/day	Multiple Sclerosis	Phase 3
NCT02322697	Effect of Carnitine on Uremic Cardiomyopathy	1000 mg/dialysis	Disorder of Fatty Acid Metabolism	Not Applicable
NCT00351234	Carnitine Levels and Carnitine Supplementation in Type I Diabetes	100 mg/kg	Diabetes Mellitus, Type I|Hypoglycemia	Not Applicable
NCT03907592	Effect of Carnitine tartrate Supplementation and Resistance Training on Skeletal Muscle Function	1000 mg/day	Sarcopenia	Not Applicable
NCT01278693	Effect of Oral l-carnitine Supplement on Lipid Profile, Anemia, and Quality of Life of Patients	1000 mg/day	Complication of Hemodialysis	Phase 2
NCT01665092	Rapid Administration of Carnitine in sEpsis	6000–18000 mg/day	Septic Shock	Phase 2
NCT00227266	Valproic Acid and Carnitine in Patients With Spinal Muscular Atrophy	1000–10000 mg/day	Spinal Muscular Atrophy	Phase 2
NCT00079599	l-Carnitine to Treat Fatigue in AIDS Patients	500–3000 mg/day	HIV Infections|AIDS	Phase 2
NCT01819701	l-carnitine and Coenzyme Q10 in Relation to the Oxidative Stress, Antioxidant Enzymes Activities, Inflammation, and the Risk of CAD	1000–2000 mg/day	Coronary Artery Disease	Phase 2|Phase 3
NCT00091169	Levocarnitine in Treating Fatigue in Cancer Patients	500–1000 mg/day	Fatigue	Phase 3
	**Combined Metabolic Cofactors**			
**NCT03838822**	Kinetics of Metabolic Cofactors (serine, NR, carnitine and NAC) in NAFLD	Serine: 20 g/day NAC: 5 g/day Carnitine: 3 g/day NR: 1 g/day	Healthy	Phase 1
EudraCT_2018-000894-59	Supplementation of Metabolic Cofactors (serine, NR, carnitine and NAC) in treatment of NAFLD	Serine: 12.35–24.7 g/day NAC: 2.55–5.1 g/day Carnitine tartrate (%73Carnitine): 3.73–7.46 g/day NR: 1–2 g/day	NAFLD	Phase 2
NCT0XXXXX	Supplementation of Metabolic Cofactors (serine, NR, carnitine and NAC) in treatment of NAFLD	Serine: 12.35–24.7 g/day NAC: 2.55–5.1 g/day Carnitine tartrate (%73Carnitine): 3.73–7.46 g/day NR: 1–2 g/day	Parkinson Disease & Alzheimer Disease	Phase 2

## Supplementary Materials

The following are available online at https://www.mdpi.com/2072-6643/11/7/1578/s1, Table S1: The dosages of the Nicotinamide Riboside, L-serine, L-carnitine and N-acetyl-L-cysteine used in previous human trials. The data retrieved from https://clinicaltrials.gov.
